# A phylogenomic analysis of the *Actinomycetales mce *operons

**DOI:** 10.1186/1471-2164-8-60

**Published:** 2007-02-26

**Authors:** Nicola Casali, Lee W Riley

**Affiliations:** 1 University of California at Berkeley, School of Public Health, 140 Warren Hall, Berkeley, CA 94720, US

## Abstract

**Background:**

The genome of *Mycobacterium tuberculosis *harbors four copies of a cluster of genes termed *mce *operons. Despite extensive research that has demonstrated the importance of these operons on infection outcome, their physiological function remains obscure. Expanding databases of complete microbial genome sequences facilitate a comparative genomic approach that can provide valuable insight into the role of uncharacterized proteins.

**Results:**

The *M. tuberculosis mce *loci each include two *yrbE *and six *mce *genes, which have homology to ABC transporter permeases and substrate-binding proteins, respectively. Operons with an identical structure were identified in all *Mycobacterium *species examined, as well as in five other *Actinomycetales *genera. Some of the *Actinomycetales mce *operons include an *mkl *gene, which encodes an ATPase resembling those of ABC uptake transporters. The phylogenetic profile of Mkl orthologs exactly matched that of the Mce and YrbE proteins. Through topology and motif analyses of YrbE homologs, we identified a region within the penultimate cytoplasmic loop that may serve as the site of interaction with the putative cognate Mkl ATPase. Homologs of the exported proteins encoded adjacent to the *M. tuberculosis mce *operons were detected in a conserved chromosomal location downstream of the majority of *Actinomycetales *operons. Operons containing linked *mkl*, *yrbE *and *mce *genes, resembling the classic organization of an ABC importer, were found to be common in Gram-negative bacteria and appear to be associated with changes in properties of the cell surface.

**Conclusion:**

Evidence presented suggests that the *mce *operons of *Actinomycetales *species and related operons in Gram-negative bacteria encode a subfamily of ABC uptake transporters with a possible role in remodeling the cell envelope.

## Background

**Figure 1:**
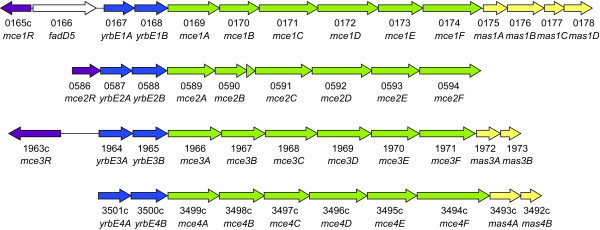
**Schematic representation of the *M. tuberculosis *H37Rv mce loci**. Proximal transcription regulators are colored in purple, *yrbE *genes in blue, *mce *genes in green, and genes encoding 'conserved *mce*-associated proteins' in yellow [44].

A putative *Mycobacterium tuberculosis *virulence gene, named *mce1A*, was originally identified because its expression in *Escherichia coli *enabled this noninvasive bacterium to enter mammalian epithelial cells [1]. Sequencing of the *M. tuberculosis *genome revealed that *mce1A *(Rv0169) was part of an operon that encoded eight putative membrane-associated proteins: YrbEA-B, MceA-F [2,3]. This operon is present four times in the *M. tuberculosis *genome (*mce1*-*4*). Homologs of the genes adjacent to the *mce1 *locus, Rv0175-Rv0178, are located downstream of the *mce3 *and *mce4 *gene clusters (Figure 1) [3].

Continued interest in the function of the *M. tuberculosis mce *operons stems from reports of the profound effect of disruption of *mce *operons on growth and virulence of the mutant strains in mice. Shimono *et al*. [4] showed that an *mce1 *mutant was hypervirulent when inoculated intravenously into BALB/c mice. In the first few weeks of infection, the mutant strain multiplied more rapidly than wild-type in the mice's lungs, spleen and liver. Surprisingly, Gioffre *et al*. [5] found that a *yrbE1B *mutant grew faster than wild-type in the lungs and spleens of BALB/c mice inoculated via the peritoneum, but more slowly in mice infected through the tracheal route. Sassetti and Rubin [6] reported that in competitive mixed infections *mce1 *mutants exhibited a growth defect in the spleens of intravenously-infected C57BL/6J mice after one week of infection. Although the exact cause of these apparently disparate phenotypes remains to be established, the observations suggest that the fate of *mce1 *mutants *in vivo *is determined by the prevailing immunological environment experienced during the first few weeks of infection.

Both *mce2 *and *mce3 *mutants replicated slower than wild-type in BALB/c mice infected via either the trachea or peritoneum [5]; however, neither mutant demonstrated a significant growth defect in competitive mixed infections [6]. In co-infected C57BL/6J mice, an *mce4 *mutant was attenuated relative to wild-type after two to four weeks infection, whilst an *mce1*-*mce4 *double mutant exhibited further attenuation, indicating that the *mce *operons perform non-redundant roles during infection [7].

The similarity of the YrbE and Mce proteins with ATP-binding cassette (ABC) transporter permeases and substrate-binding proteins, respectively, has been noted previously [8,9]. ABC transporters couple the energy released by ATP hydrolysis to the translocation of a substrate across a membrane. Members of the ABC transporter family are ubiquitous in living organisms and comprise one of largest superfamilies known [10].

A functional ABC transporter system minimally contains two cytoplasmic nucleotide-binding ATPase domains and two transmembrane channel-forming permease domains. These components can be homo- or heterodimers and may be encoded on separate or fused polypeptides. Both eukaryotes and prokaryotes contain ABC exporters, whereas importers have been identified only in prokaryotes. Importers additionally require substrate-binding proteins (SBPs) that provide specificity and high-affinity. Typically, SBPs are periplasmic in Gram-negative bacilli and lipoproteins in Gram-positive bacilli [11]. SBPs share a two-lobed quaternary structure with a central cleft that undergoes a large conformational change upon ligand-binding, promoting close interaction with the cognate permease. This results in hydrolysis of ATP, which energizes translocation of the substrate [12]. In Gram-negative bacteria, SBP-dependent importers also usually require porins or specific receptors to facilitate transport across the outer membrane [11].

The genes encoding the ATPase, permease and SBP components of an ABC transporter are often contiguous in the genome and comprise an operon. Phylogenetic clustering of the individual transporter components is almost always concordant, indicating that the operons have arisen from a common ancestral transporter with minimal shuffling of constituents. In addition, sequence similarity shows good correlation with substrate specificity [13-15].

The ATPase is the most conserved component of the system and transporter function is frequently predicted solely on the basis of ATPase orthology [10,15]. These proteins contain a homologous region, of 200 amino acids, with several characteristic motifs: Walker A and B motifs in the nucleotide-binding fold [16], as well as a signature motif found only in ABC transporter-associated, or 'traffic', ATPases [17].

The permease components and SBPs have limited primary sequence similarity, and thus their identification is not facile. They are typically identified in genome sequences by their proximity to ATPases and, for permeases, possession of predicted transmembrane regions [18-20]. The inference of function through sequence comparison has traditionally relied upon similarity to close homologs of known function. The advent of the genomic age has provided invaluable new methods for the elucidation of roles of proteins with unknown function. Non-homology-based methods of genome comparison use patterns of domain fusion [21], conserved chromosomal location [22], and phylogenetic profiles [23], to predict functional interactions between proteins. In addition, the availability of hundreds of complete genome sequences permits the reliable identification of orthologs, operationally-defined as reciprocal best hits [24], enabling more precise functional prediction than sequence similarity alone. These methods are non-redundant and their application can facilitate deduction of specific function [25]. Here we endeavor to further understand the function of the *M. tuberculosis mce *operons, and assess the likelihood that they encode ABC transporters, through sequence and genome comparisons, database mining and the application bioinformatic methods.

## Results

### Distribution of *mce *operons in *Actinomycetales*

Perusal of databases of conserved domains, such as InterPro [26], Pfam [27] and TIGRFAM [28], constitutes a simple method for the identification of homologous proteins. The *M. tuberculosis *H37Rv genome encodes 24 Mce proteins, each of which contains a conserved domain of 304 amino acids defined by the TIGRFAM family: TIGR00996 (IPR005693). Members of this family are confined to the Order *Actinomycetales*. The corresponding Pfam family, PF02470 (IPR003399), describes a 98 amino acid sub-region of the Mce domain that is more widely distributed (see below). The *mce *genes in *M. tuberculosis *are clustered in groups of six; each cluster is preceded by two copies of a gene termed *yrbE *(Figure 1). Databases of conserved domains group the YrbE proteins into a family called DUF140 (domain of unknown function). Pfam defines the family by a region approximately 150 amino acids long (PF02405; IPR003453). The corresponding TIGRFAM family (TIGR00056) describes a subfamily of DUF140, but excludes the mycobacterial homologs based on a stated extreme divergence at the amino end. For the sake of clarity, we refer to a cluster of genes encoding two YrbE and six Mce proteins as an '*mce *operon'.

To assess the distribution of *mce *operons in completed and draft assemblies of genomes of members of the Order *Actinomycetales*, we surveyed the annotation of predicted proteins for members of Pfam families PF02470 and PF02405 (Table 1). The proteomes of all 10 *Mycobacterium *species examined contained Mce proteins. The number varied from 6 in *Mycobacterium leprae *up to 66 in *Mycobacterium vanbaalenii*. Other genomes containing *mce *genes belonged to species of *Nocardia*, *Janibacter*, *Nocardiodes*,* Amycolatopsis *and *Streptomyces*. Mce homologs were absent from 18 *Actinomycetales *genomes, notably including those of the four sequenced *Corynebacterium *species. DUF140 proteins were found encoded within all *Actinomycetales *genomes that contain *mce *genes and were absent from all genomes that do not contain *mce *genes. Other completely sequenced genomes of species belonging to the Class *Actinobacteria*, namely *Rubrobacter xylanophilus*, *Symbiobacterium thermophilum *and *Bifidobacterium longum*, did not contain either Mce or DUF140 homologs.

**Figure 2:**
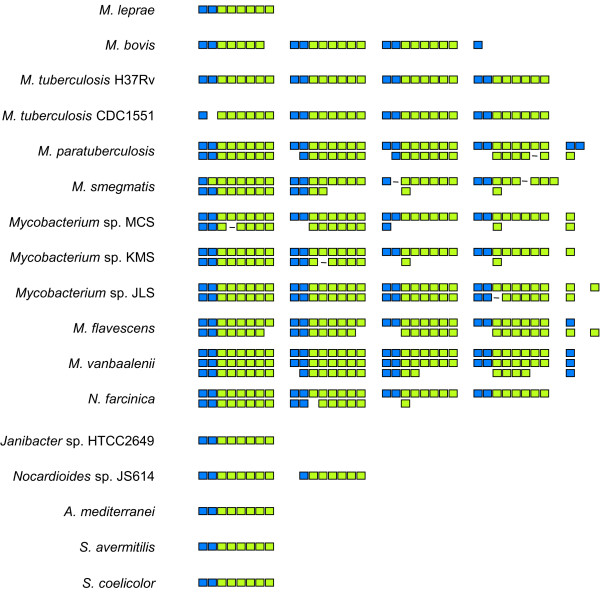
**Schematic representation of the organization of *mce *loci in *Actinomycetales *genomes**. Genes encoding proteins belonging to Pfam family PF02470 (Mce) are depicted as green boxes, and to family PF02405 (DUF140) as blue boxes. Dashes indicate gaps in gene numbering.

Examination of the genomic location of the Mce and DUF140 homologs revealed that the *mce *genes were almost always found clustered in groups of six, located downstream from a pair of DUF140 genes (Figure 2).

### Identification of *mce*-like operons in Gram-negative bacteria

A 98 amino acid sub-region of Mce family proteins, termed the 'Mce-like' domain (PF02470), is widely distributed in Gram-negative bacteria and has also been found encoded in plant genomes. No Mce-like domains have been identified in any Archeael or low GC-content Gram-positive bacterial genomes.

**Figure 3:**
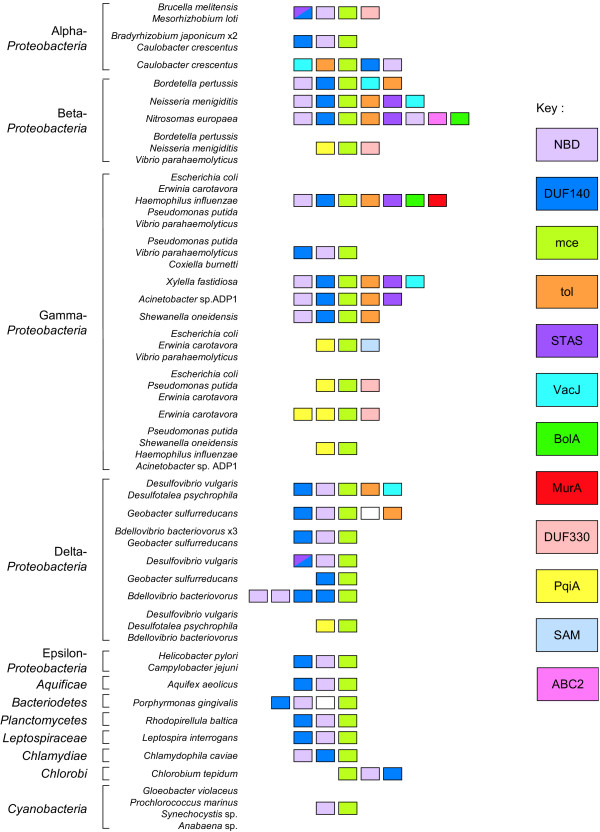
**Conserved proteins encoded in the neighborhood of *mce *genes in Gram-negative bacteria**. Coloring reflects conserved domains identified in the key. Protein families shown are: NBD, an ABC transporter ATPase (IPR003439); DUF140 (IPR003453); Mce (IPR003399); Tol, a Ttg2 toluene tolerance protein (IPR008869); STAS, a domain found in sulfate transporters and anti-sigma factor antagonists (IPR002645); VacJ, a lipoprotein of unknown function (IPR007428); BolA, a possible regulator induced by stress (IPR002634); MurA, UDP-N-acetylglucosamine-1-carboxyvinyltransferase (IPR005750); DUF330 (IPR005586); PqiA, an integral membrane protein inducible by superoxide generators (IPR007498); SAM, an S-adenosyl methionine binding methyltransferase (IPR000051); and ABC2, an ABC-2 type permease (IPR013525).

Genes with related functions are frequently encoded within operons and thus found clustered in the genomes of prokaryotes [22]. We investigated the gene neighborhoods of selected *mce*-like genes with the aim of obtaining clues regarding the biological role of proteins of this family (Figure 3). The Mce-like proteins in Gram-negative bacteria were frequently found clustered in the genome with a DUF140 family protein and an ATPase homolog (IPR003439) in an arrangement typical of an ABC transporter system [11]. The three components were found encoded in any order and in some instances either the DUF140 or ATPase homolog was duplicated. In a number of γ-*Proteobacteria *the ATPase-DUF140-Mce cluster was encoded in a conserved genomic region that included a Tol protein (IPR008869), a STAS domain protein (IPR002645) and MurA(IPR005750), the product of which catalyses the first step of murein biosynthesis. Like Mce domains, Tol proteins have homology to SBPs [29]; the presence of SBPs indicates that these operons encode substrate uptake transporters. Aravind and Koonin suggested that the nucleotide-binding activity of STAS domains, found in sulfate transporters, could regulate uptake in response to intracellular ATP or GTP concentrations [30]. Several DUF140 proteins that are N-terminally fused to STAS domains have been identified [31], implying a functional linkage between these two proteins in the *mce *operons [21]. The Mce transporter clusters were also frequently found associated with homologs of a surface-exposed lipoprotein VacJ (IPR007428), and the morpho-protein BolA (IPR002634).

The Mce homologs in these putative transporter operons each contain a single 98 amino acid Mce-like domain. Many proteobacterial genomes additionally contain Mce homologs, sometimes annotated as PqiB, that contain 2–7 copies of the Mce-like domain and are usually associated with a PqiA family protein (IPR007498) of unknown function. The *E. coli pqiAB *operon is induced by treatment with the model superoxide generator, paraquat [32].

### Mce-associated ATPases

Since ABC transporters absolutely require an ATPase to provide the energy required for substrate translocation, the genes neighboring the *Actinomycetales mce *operons were inspected for ATPase homologs (IPR003439). Although none of the mycobacterial *mce *operons neighbors an ATPase, a candidate gene was identified immediately upstream of a single *mce *operon in the genome of every non-mycobacterial *Actinomycetales *species that possesses *mce *genes (Table 2). BLASTP analyses demonstrated that the corresponding protein sequences were reciprocal best hits with the *mce*-linked ATPases in Gram-negative bacteria, indicating orthology [24]. A phylogenetic analysis of ABC transporter ATPases reported by Dassa and Bouige groups these *Actinomycetales *and Gram-negative bacterial ATPases into a family termed Mkl [8].

**Figure 4:**
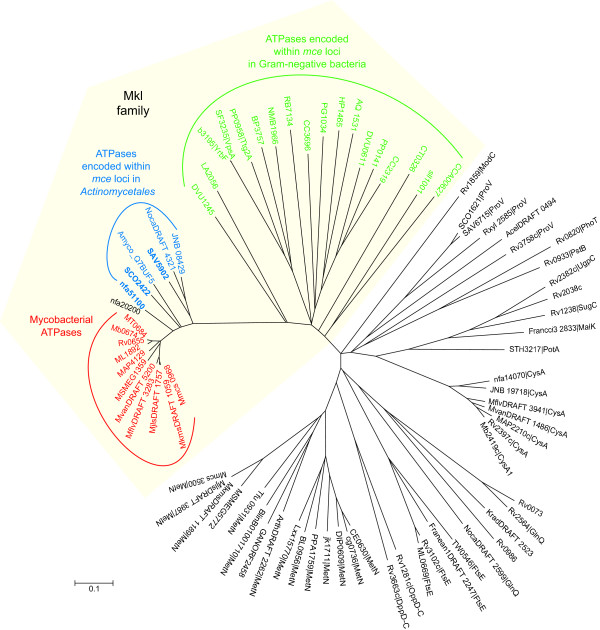
**Phylogenetic tree showing relationship between *mce*-linked ATPases and mycobacterial orthologs**. ATPases encoded within *mce *operons in *Actinomycetales *species are colored blue; those in Gram-negative bacterial *mce *operons are colored green. The sequences most similar to nfa51100, SAV5902 and SCO2422 (indicated in bold), in the *Actinomycetales *genomes listed in Table 1, were identified by BLASTP searches and included in the tree. All of the best hits from mycobacterial species cluster within the Mkl family and are colored red. For comparison, sequences of all *M. tuberculosis *H37Rv ATPases of ABC uptake transporters were included [20]. All of the top hits from *Actinomycetales *that do not possess *mce *operons are rooted among these non-*mce*-linked ATPases, as are all of the second hits from mycobacterial species. ORFs are designated by (UniProt gene name | protein name).

The sequences of the *N. farcinica *and *Streptomyces mce*-linked ATPases (nfa51100, SAV5902 and SCO2422) were used as BLASTP queries in order to identify additional Mkl-like ATPases. The best hits from each of the completed *Actinomycetales *genomes (Table 1) were retrieved for further evaluation. Phylogenetic analysis of the protein sequences revealed that each *Mycobacterium *species contained a single ATPase that clustered with the Mkl family, providing strong evidence of orthology (Figure 4, Table 2). In addition, a paralog was identified in the *N. farcinica *genome (nfa20200); this ORF is annotated in The Institute of Genome Research (TIGR) database as MetN, a D-methionine ABC transporter ATPase, but it does not cluster with other putative MetN orthologs (Figure 4).

Comparison of the most closely related ORFs in other *Actinomycetales *revealed that only those genomes that contained *mce *operons possessed an orthologous ATPase (Figure 4). Congruency of the phylogenetic profiles of the Mkl ATPases with YrbE and Mce proteins provides further evidence of functional association [23].

Each of the *mce*-linked ATPases and mycobacterial orthologs contain the conserved Walker A and B motifs required for ATP binding, as well as the ABC transporter family signature (LSGGQ) with no more than one mismatch [16,33]. In a published analysis of *M. tuberculosis *ABC transporters, the putative Mce ATPase, Rv0655, segregated with importers but did not fall into any of the previously described families with known substrates [20]. Similarly, in a more expansive study, the Mkl family ATPases fell into the SBP-dependent importer clade, but clustered separately from those with established specificity [8].

The mycobacterial Mkl ATPases and nfa20200 and are not genomically located near any other ABC transporter components and appear to be transcriptionally-isolated. The *M. leprae *ortholog is located adjacent to RNA polymerase *rpo *genes leading to speculation that this ATPase was involved in ribonucleotide uptake [34]. Consequently, Mkl ATPases are sometimes annotated as ribonucleotide uptake systems.

### The Mce proteins

Comparison of the amino acid sequences of the Mce proteins encoded in the genomes of *Mycobacterium bovis *and the *M. tuberculosis *strains H37Rv, CDC1551 and 210, revealed that each of the *M. tuberculosis *genomes contained 24 Mce ORFs, whilst, as noted previously, the *mce3 *operon is deleted in *M. bovis *[35]. A number of genes were found to contain frameshift mutations: *mce1F *in strain 210; *mce2B *in strains H37Rv and CDC1551; *mce2C *in strain CDC1551; and *mce2D *and *mce2E *in *M. bovis*. The truncated ORFs thus conspicuously clustered within the *mce2 *operon.

A non-redundant set of Mce proteins from the genomes of *M. tuberculosis*, *M. bovis*, *M. leprae*, *Mycobacterium avium *subsp. *paratuberculosis *(*M. paratuberculosis*), *Mycobacterium smegmatis*, *N. farcinica*, *S. coelicolor *and *S. avermilitis *were selected for further analysis. Examination of the genomic regions of partial operons revealed the presence of several additional putative Mce homologs that were included in this analysis (Table 3).

**Figure 5:**
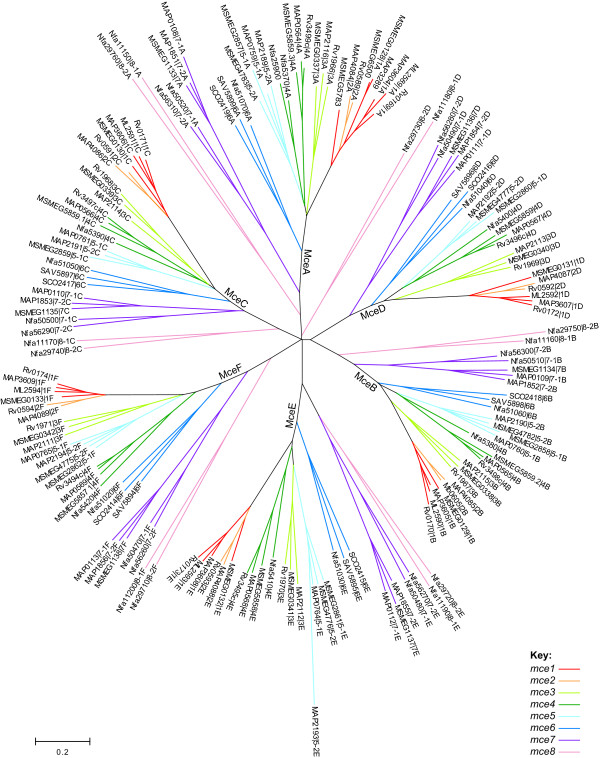
**Phylogenetic tree of *Actinomycetales *Mce proteins**. A non-redundant set of Mce protein sequences were aligned and an unrooted neighbor-joining tree was computed by MEGA. Coloring corresponds to the classification scheme specified in Table 3. ORFs are designated by [gene locus name | operon number (1–8) and gene position (A-F)]. Where operon orthology cannot be inferred, operons are designated: -1, -2.

Multiple alignment and phylogenetic analysis of the Mce homologs revealed six distinct branches, which corresponded exactly to the encoding genes in the respective operons (that is *mceA*-*F*; Figure 5). Within each of the six major branches, the clustering of sequences was essentially the same. This pattern indicates that each *mce *gene cluster duplicated from an ancestral operon that contained six *mce *genes and that no shuffling between or within operons has occurred.

We have classified the operons as *mce1*-*8 *according to the clustering observed (Table 3). The *mce1 *and *mce2 *operons are the most closely related and duplication may have occurred after divergence of the fast- and slow-growing mycobacteria, since *M. smegmatis *contains a single copy. Although the orthology of the *M. smegmatis *operon cannot be deduced from the phylogenetic tree, we infer from synteny that it is orthologous to the *M. tuberculosis mce1 *operon. Thus, *mce1 *is the sole operon that is found in all, and in only, the *Mycobacterium *species examined. The *Streptomyces *operons fall into a cluster, termed *mce6*, that does not contain any mycobacterial orthologs, but is found in *N. farcinica*. The Mkl-like ATPase is located upstream of *yrbEA6 *in all three of these operons. In several cases operon orthology could not be deduced from the branching pattern observed, presumably due to recent duplication events. Thus, it appears that *M. paratuberculosis *and *M. smegmatis *possess two copies of the *mce5 *operon; *M. paratuberculosis *and *N. farcinica *have two copies of the *mce7 *operon; and *N. farcinica *has two copies of the *mce8 *operon. The *M. paratuberculosis *Mce5E protein (MAP2193) seems to have diverged significantly from its paralog (MAP0764); examination of the encoding sequences revealed that this is a consequence of a 40bp deletion, which results in a frameshift of the N-terminal 120 amino acids.

One and two extra copies of Mce1A were found in *M. paratuberculosis *(MAP3289) and *M. smegmatis *(MSMEG5783, MSMEG6500), respectively; whilst *N. farcinica *contained a second copy of Mce4A (nfa25900). Each of the encoding genes appeared to be transciptionally isolated, with the exception of MSMEG5783, which is located within a four-gene operon that includes pyridoxamine 5-phosphate oxidase and a putative lipoprotein.

**Figure 6:**
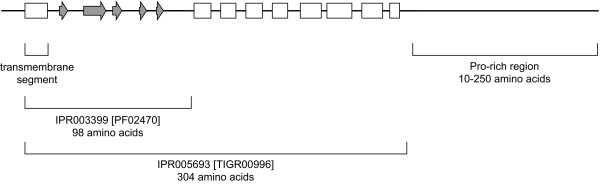
**Illustration of conserved regions and predicted secondary structure of *Actinomycetales *Mce proteins**. Six separate alignments of the Mce proteins (A-F) listed in Table 3 were submitted to JPred and the consensus secondary structure prediction estimated manually. White boxes represent α-helices and grey arrows β-strands. The C-terminal proline-rich region had low complexity and varied in length from 10–250 amino acids. Signal sequences were identified by SignalP and lipid attachment sites matched the ProSite motif PS00013.

Secondary structure predictions, through the JPred server, revealed the consensus structure of the conserved Pfam region folded into five β-strands; the central region of *Actinomycetales *Mce proteins, included in the conserved TIGRFAM region, contains eight α-helices. The C-terminal region varies in length from 10–250 amino acids, has predicted low complexity and is rich in proline residues (Figure 6). Length is not conserved within the six homologous families, with the exception of the MceB proteins in which the C-terminal region is 30–50 amino acids in all cases. On average the MceA and MceF proteins are the longest. An RGD motif was identified in the C-terminal tail of 16 (of 27) MceE sequences. This motif is known to bind integrins, as well as C2 domains [36,37].

Each of the Mce proteins contained a hydrophobic stretch at the N-terminus, likely to be a transmembrane helix. Using a neural network trained on Gram-positive bacteria the program SignalP predicted a signal peptide cleavage site for 98 of 161 of these proteins [38]. There was no correlation between prediction of secretion and Mce-type (A-F) or bacterial species. Although the Mce anchor regions frequently contained a pair of arginine residues, characteristic of Twin-arginine transporter (Tat) motifs, few (12 of 161) are recognized as Tat substrates [39]. A lipoprotein attachment site (PS00013) was present in 22 of 27 MceE proteins. The highly conserved operon structure containing six *mce *genes suggests that they associate to form a heteromeric complex [22,40], which is therefore likely to remain tethered to the cell membrane even if some proteins are cleaved. Indeed, Mce1A-1F have been shown to localize to the cell envelope of *M. tuberculosis *[4].

### The YrbE proteins

Unlike the Mce proteins, the amino acid sequences of YrbE orthologs in the *M. tuberculosis *strains H37Rv, CDC1551 and 210, as well as *M. bovis*, were found to be >99.5% identical in all cases. The sequences of the YrbE proteins associated with the *mce *gene clusters of *M. tuberculosis*, *M. leprae*, *M. paratuberculosis*, *M. smegmatis*, *N. farcinica*, *S. coelicolor *and *S. avermilitis *were selected for further analysis. In several cases the ORF downstream of *yrbEA *was either not annotated or annotated in the reverse direction; however, translation of the genomic sequence revealed a YrbEB homolog encoded in the expected direction (Table 3).

**Figure 7:**
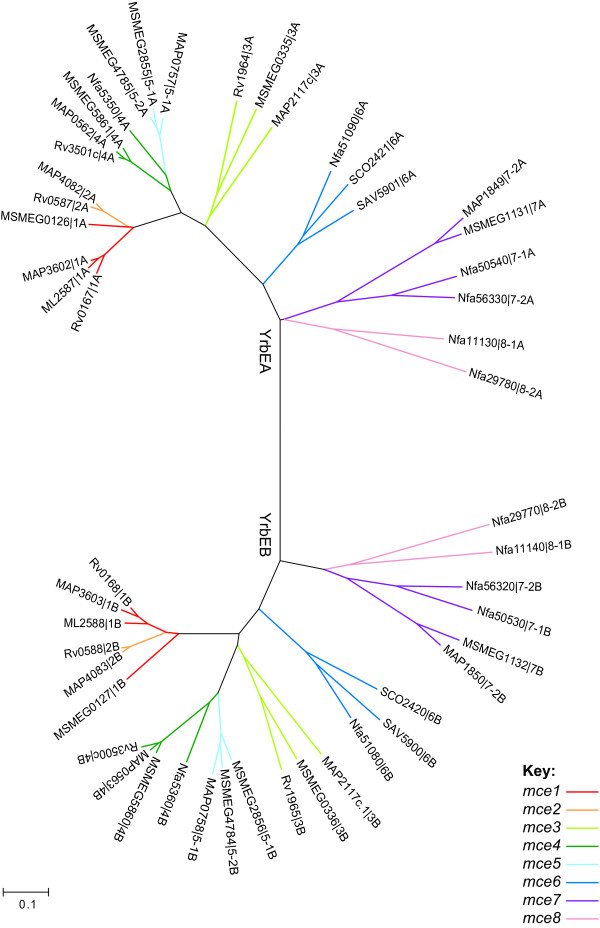
**Phylogenetic tree of *Actinomycetales *YrbE proteins**. A non-redundant set of YrbE protein sequences were aligned and an unrooted neighbor-joining tree was computed by MEGA. Coloring corresponds to the classification scheme specified in Table 3. ORFs are designated by [gene locus name | operon number (1–8) and gene position (A, B)]. Where operon orthology cannot be inferred, operons are designated: -1, -2.

Phylogenetic analysis showed deep branching between the YrbEA and YrbEB sequences (Figure 7). Within each clade the clustering of sequences was almost identical demonstrating that the *yrbEA*-*yrbEB *genes have evolved as a pair. The clustering was comparable to that seen in the Mce protein tree, with members of the *mce1*/*2 *and *mce3 *to *mce8 *operons easily distinguishable. Thus, it appears that all of the operons examined evolved from a common ancestral eight-gene cluster without shuffling of genes within or between operons.

**Figure 8:**
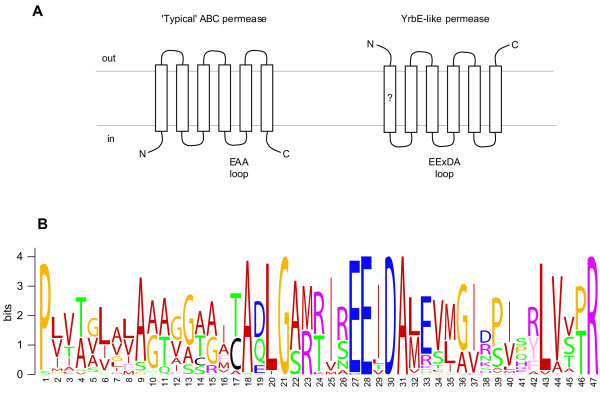
**Predicted topology and conserved sequence motif of *Actinomycetales *YrbE proteins**. (A) The consensus topology prediction of Actinomycetales YrbE proteins analysis is shown compared to that of a typical ABC permease [42]. (B) WebLogo illustration of the conserved YrbE EExDA sequence motif identified through MEME analysis.

ABC permeases typically contain six transmembrane segments with the C-terminus located on the cytoplasmic side of the membrane [11]. The consensus TMHMM-predicted structure of *Actinomycetales *YrbE homologs found in *mce *operons suggests the presence of five or six transmembrane helices with the C-terminus outside (Figure 8a). The presence of the N-terminal transmembrane helix was equivocal, and therefore the N-terminus may be cytoplasmic or outside. Further topological predictions using the programs HMMTOP and TopPred confirmed this model, but were unable to verify or refute the existence of the N-terminal transmembrane segment.

Dassa and colleagues [41,42] have described a highly-conserved sequence, the EAA motif, in the final cytoplasmic loop of some SBP-dependent ABC permeases that is proposed to interact with the cognate ATPase [43]. Examination of the multiple alignment of YrbE proteins revealed a conserved sequence motif located in the penultimate cytoplasmic loop. The consensus deduced from 50 *Actinomycetales *YrbEA and YrbEB sequences is shown in Figure 8b. Alignment of Gram-negative bacterial DUF140 proteins revealed that this region was highly conserved in all family members. The consensus sequence we have deduced does not appear to be homologous to the published motifs, but does contain the common invariant glycine residue and is predicted to adopt the typical α-helical structure [42]. The consensus 47 amino acid YrbE sequence, that we have termed the EExDA motif, was able to specifically retrieve *Actinomycetales *and Gram-negative DUF140 proteins from the National Center for Biotechnology Information (NCBI) microbial proteomes database.

In one case (*Rhodopirellula baltica*, RB3287) a DUF140 domain is fused to an ABC ATPase domain providing evidence that the function of DUF140 proteins requires ATP hydrolysis [21].

### The Mas proteins

The four genes downstream of the *M. tuberculosis mce1 *operon, as well as two each downstream of the *mce3 *and *mce4 *operons, are annotated in TubercuList [44] as 'conserved mce-associated proteins' (herein termed Mas). The *mce1 *operon transcript has been empirically demonstrated to include the associated *mas *genes (Rv0175-78) [45]. Examination of a multiple alignment of the protein sequences revealed that they were not conserved along their entire length but shared a similar C-terminal region of approximately 160 amino acids. Pairwise sequence identity scores, generated by ClustalX, for the conserved region ranged from 12 to 25%.

To determine whether homologous domains were present in other genomes, we used each of the eight Mas C-terminal sequences as a PSI-BLAST query against the NCBI non-redundant database. A total of 137 sequences were retrieved; of these, 124 sequences were hit by all eight query sequences, and all 137 were hit by more than two queries. The proteins identified belonged to six genera: *Amycolatopsis*, *Janibacter*, *Mycobacterium*, *Nocardia*, *Nocardiodes *and *Streptomyces*. Thus, the phylogenetic profile for the putative Mas homologs in *Actinomycetales *genera exactly matches that of the Mce, DUF140 and Mkl proteins. Mas homologs in the *M. smegmatis *genome, which was not covered by the NCBI database, were identified by exhaustive BLAST querying of the TIGR proteome. Nineteen putative Mas homologs were thus identified (P < 0.00001).

Sequences of the putative Mas domain containing proteins from *M. tuberculosis*, *M. leprae*, *M. paratuberculosis*, *M. smegmatis*, *N. farcinica*, *S. avermitilis *and *S. coelicolor *were selected for further analysis. This resulted in a set of 66 sequences (including one hybrid sequence, MAP2107/9c, that has been disrupted by a transposase).

The Mas domain genes were typically found in pairs (58 of 66) and the majority (43 of 66) were encoded downstream of, and in the same direction, as *mce *genes (Table 4). Putative orthologs of each of the eight *M. tuberculosis mce *operon-associated *mas *genes were identified in the corresponding positions of those genomes carrying orthologous operons. Each of the *mce7 *operons had a single Mas protein encoded downstream. The *mce6 *operons of *N. farcinica *and *S. avermilitis *contained two *mas *genes, while the corresponding *S. coelicolor *operon carried four. In *M. paratuberculosis*, a pair of *mas *homologs was located in the regions both upstream and downstream of the *mce5 *operon, but transcribed from the opposite strand (MAP0750-51c, MAP0767-68c). The 23 non-*mce *operon-associated Mas homologs were generally located in pairs in isolated operons. An exception was Rv2390c, which TIGR predicts is part of a three-gene operon including a resuscitation promoting factor (*rpfD*, Rv2389c) and an Fe-S enzyme involved in porphyrin biosynthesis (*hemN*, Rv2388c).

The Mas region is not currently recognized as a conserved domain in the databases. However, within this region, InterPro recognized a lipocalin family motif (IPR002345) in Rv3492c, and a partial C2 domain signature (IPR000008) in Rv0199 and ML2614. Notably, the corresponding Pfam families (PF00061 and PF00168) did not include these sequences as members. Nonetheless, it may be worthy of mention that the lipocalin and C2 domains share a lipid-binding function, as well as an eight-stranded anti-parallel beta sandwich structure [46,47].

**Figure 9:**
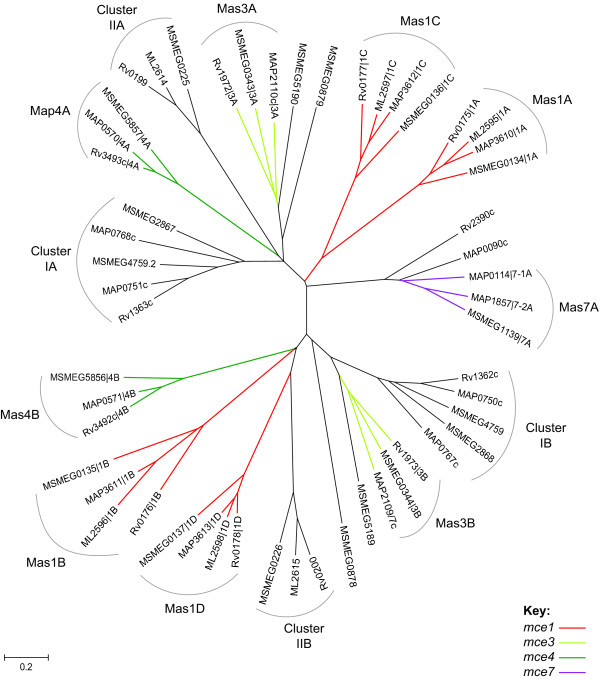
**Phylogenetic tree of mycobacterial Mas domain sequences**. The conserved Mas domains of mycobacterial proteins listed in Table 4 were aligned and an unrooted neighbor-joining tree was computed by MEGA. Coloring corresponds to the classification scheme specified in Table 3. ORFs are designated by [gene locus name | operon number (1, 3, 4, 7) and gene position (A-D)]. Where operon orthology cannot be inferred, operons are designated: -1, -2.

The majority of pairwise identity scores for the 66 Mas domains were 10–20%. This low level of sequence similarity resulted in multiple sequence alignments that were extremely sensitive to input parameters. Exclusion of the 13 non-mycobacterial sequences produced a much more robust alignment. A phylogenetic tree generated from this alignment is shown in Figure 9. Examination of the tree revealed that the Mas proteins encoded by the first and second genes in each pair formed phylogenetically distinct clusters. The Mas proteins encoded adjacent to *mce *operons were not separated from the non-*mce *associated Mas proteins. The *M. leprae*, *M. paratuberculosis *and *M. smegmatis *Mas proteins associated with the *mce1*, *mce3 *and *mce4 *operons are clearly orthologs of those in the corresponding genomic positions in *M. tuberculosis*. The *mce7*-associated Mas proteins also cluster together. Several pairs of non-*mce *associated Mas homologs were conserved between mycobacterial species (Figure 9; Cluster I and Cluster II).

The mycobacterial *mce*-associated Mas orthologs have greater than 50% pairwise identity. In contrast, the *Nocardia *and *Streptomyces mce6*-associated Mas proteins are highly divergent (15–20% identity). This suggests that, unlike the *mce *and *yrbE *genes, the *mas *genes have either diverged more rapidly or were independently recruited to the operons.

Comparison of JPred secondary structure predictions for orthologous clusters revealed the consensus structure of the conserved domain was α_1_α_2_α_3_α_4_β_1_β_2_β_3_β_4_. Prediction of transmembrane helices indicated that all 66 protein sequences harbored a transmembrane segment located about 140–180 amino acids from the C-terminus and corresponding to α_1_. Topology prediction programs, TMHMM, HMMTOP and TopPred, suggested the C-terminus was extracellular for 41, 56 and 42, of the 66 submitted sequences, respectively. In no case did all three programs predict an extracellular N-terminus for a single protein. Thus, it seems likely that all N-termini are intracellular, while the C-terminal Mas domains are located on the external side of the cytoplasmic membrane.

**Figure 10:**
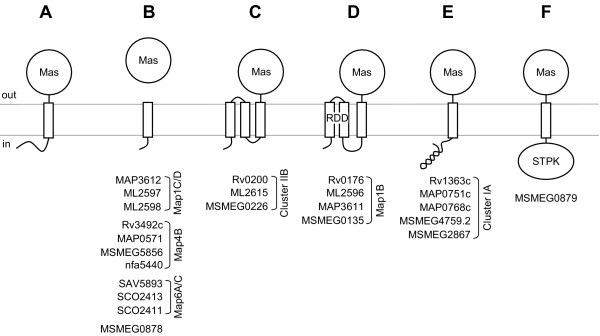
**Representative architectures of Mas domain-containing proteins**. Membrane topology predictions for the 66 Mas proteins listed in Table 4 indicated that the conserved domain was located on the extracellular side of the cytoplasmic membrane. The Mas domain was predicted to remain anchored in the majority of proteins (A), but cleaved in eight (B). Three transmembrane segments were identified in seven proteins and four of these were classified as RDD domains (C, D). Five proteins contained an N-terminal coiled-coil region (E), and one, a serine-threonine protein kinase domain (STPK; F).

The length of the N-terminal region preceding the Mas domain ranged from 7 to 325 amino acids. In the majority of proteins in which the N-terminal segment was less than 30 amino acids (11 of 16), α_1 _was predicted to be a signal peptide by SignalP (Figure 10). Consensus topology predictions indicated that the four Mas1B orthologs and three Cluster IIB proteins contained two N-terminal transmembrane helices (oriented in-out, out-in). In the Mas1B orthologs, the two N-terminal transmembrane segments correspond to an RDD domain (IPR010432). Examination of a multiple alignment revealed that although *M. smegmatis *Mas1B does not actually have the N-terminal signature RD residues, the Cluster IIB proteins do. It has been proposed that the RDD domain is involved in transport [31]; however, to date, no empirical evidence has been published to support this claim. In MSMEG0879 the 325 amino acid N-terminal region encodes a protein kinase domain (IPR000719) containing the Ser/Thr kinase active site motif (PS00108). Coiled-coils, which are known to mediate protein-protein interactions [48], were identified in the N-terminal region of each Cluster IA sequence by the Lupas COILS algorithm.

## Discussion

In this study we sought to gain insight into the function of the *M. tuberculosis mce *operons using genome comparisons and bioinformatic methods.

The YrbE and Mce proteins, encoded by the *M. tuberculosis mce *operons, have homology to the permease and SBP components of ABC transporters, respectively [29]. However, sequence similarity within these protein families is notoriously low, and confirmation that the *mce *operons encode ABC importers has required identification of the necessary cognate ATPase. Dassa and Bouige [8] have proposed that Rv0655, an ATPase named Mkl, might supply this function and here we provide substantial evidence that this is indeed the case.

Firstly, Mkl orthologs are encoded immediately upstream of the mycobacterial-like *mce *operons in species of *Nocardia*,* Janibacter*, *Nocardioides*, *Amycolatopsis *and *Streptomyces*. Secondly, orthologs of Mkl are found in all, and in only, those *Actinomycetales *species that also contain Mce and DUF140 homologs. The presence of an intact *mkl *gene in the *M. leprae *genome, which has undergone extensive reductive evolution [49], is significant in this respect. Thirdly, in Gram-negative bacteria, operons containing DUF140 and *mce *homologs invariably include the orthologous *mkl *gene. Recently, Joshi *et al*. [7] observed that in competitive mouse infections an Rv0655 mutant was attenuated relative to wild-type *M. tuberculosis*, whereas an Rv0655-*mce1 *double mutant showed no attenuation relative to the *mce1 *mutant, providing evidence that Rv0655 and the Mce1 proteins are functionally linked. It is notable that in the *Mycobacterium *species examined, the *mkl *gene is located within the genomic region that encodes the majority of ribosomal proteins; this is generally the most conserved region in prokaryotic genomes and could facilitate high level expression of *mkl *[40].

It is widely accepted that the direction of substrate transport of ABC transporters can be predicted on the basis of ATPase homology [10]. In phylogenetic analyses, Mkl ATPases fall into the importer clade [8,20]; this prediction is consistent with the proposed role of Mce proteins as SBPs, which are found exclusively in substrate import systems.

The results of topology prediction indicated that the YrbE proteins contained five to six transmembrane segments, with the C-terminal five the most conserved and the C-terminus outside. In support of this model, the periplasmic location of the C-terminus of *E. coli *YrbE has been demonstrated empirically [50]. In general, ABC permeases show the highest level of sequence similarity over the C-terminal five transmembrane regions, and this is considered to be the minimal functional unit [11]. In compiled alignments of ABC permease sequences, the most conserved region localizes to the final cytoplasmic loop [42]. This motif, termed the EAA loop, likely interacts with the cognate ATPase [43]. A highly conserved motif, predicted to localize to the penultimate cytoplasmic loop, was identified in YrbE proteins from both *Actinomycetales *and Gram-negative bacteria. We propose that this motif, named the EExDA loop, serves as the site of interaction with the putative cognate Mkl ATPase, in a manner analogous to the EAA loop.

Conservation of the 'two *yrbE *plus six *mce*' operon structure suggests that these components comprise the functional unit of the canonical *Actinomycetales *Mce transporter [22,40]. We have found that mutation of either the *yrbE1A*, *mce1A *or *mce1E *genes of *M. tuberculosis *results in undetectable levels of all the Mce1 proteins, implying that these proteins are part of a hetero-octomeric complex and its formation is necessary for stability of the Mce proteins [4] (L. Morici, personal communication). It is interesting that many *Proteobacteria *contain membrane proteins with multiple Mce domains (PqiB proteins) that could potentially interact forming a quaternary structure analogous to the putative *Acinomycetales *Mce complex. The permease components of ABC transporters, that form a channel across the cytoplasmic membrane, are frequently heterodimers; however, although present in stoichiometric excess, SBPs are generally encoded by one or two genes [11]. The presence of six SBPs is, thus far, a unique characteristic of the *Actinomycetales *Mce transporters. Using computational methods, Pajon *et al*. [51] found that the β-sheet region of eight of the *M. tuberculosis *Mce proteins contained patterns typical of transmembrane β-strands and suggested that this region could promote penetration of the outer lipid layer. Thus, it is tempting to speculate that the Mce proteins are designed to form a channel that crosses this lipid bilayer. Chitale *et al*. [52] have previously shown that Mce1A is indeed exposed on the surface of *M. tuberculosis*.

Proteins encoded downstream of three of the four *M. tuberculosis mce *operons exhibit significant sequence homology. Similarity is confined to the 160 amino acid C-terminal region, we have termed the Mas domain, that is predicted to localize to the extracellular side of the cytoplasmic membrane. In each of the *Actinomycetales *genomes examined, Mas domain proteins were found linked to the majority of *mce *operons. Mas proteins show absolute phylogenetic congruency with Mkl, DUF140 and Mce proteins in the genomes of *Actinomycetales*, providing evidence that they are involved in Mce transporter function. Given that Mas domains are not found associated with all *mce *operons, their function may not always be strictly required or they may be shared between operons. The propensity of Mas homologs to be located in pairs suggests that they form heterodimers. Such an interaction would likely keep the predicted secreted Mas proteins tethered to the cell surface. The domain architectures of the Mas proteins suggest that the conserved domain plays an accessory ligand-binding role.

Several studies have shown that the γ-proteobacterial *mce *loci play a role in determination of structural properties of the cell envelope, which in pathogenic species affects invasive activity. In *Pseudomonas putida*, a transposon insertion within the DUF140-Mce-associated *ttg2A *ATPase (PP0958) renders the cells sensitive to toluene [53]. In addition to toluene degradation and efflux, toluene tolerance is known to be mediated by increased cell membrane rigidity resulting from changes in fatty acid and phospholipid composition [54]. In *Shigella flexneri*, mutations in the *vpsABC *locus (S_3453-51), encoding an ABC transporter with the ATPase-DUF140-Mce configuration, result in a defect in intercellular spread through epithelial cell monolayers, altered colony morphology, increased sensitivity to detergent lysis and hypersecretion of both Sec-dependent and TypeIII-dependent virulence proteins [55]. Carvalho *et al*. have reported that in *Campylobacter *isolates, presence of *iamA*, the ATPase gene of the *mce *operon (Cj1646-48), correlated with an invasive phenotype [56], although, this association remains controversial [57-59]. In *Neisseria meningitidis *the *mce*-like operon, *gltT *(NMB1966-64), belongs to the GdhR regulon, which is expressed at higher levels in invasive versus commensal isolates, and is particularly elevated in hypervirulent lineages [60].

Comparable function has been attributed to the *M. tuberculosis mce1 *operon. The prototypical Mce protein, *M. tuberculosis *Mce1A, conferred invasive ability upon *E. coli *and an *M. bovis *BCG *mce1A *mutant exhibited impaired invasion of epithelial cells [1,61]. Moreover, an *M. tuberculosis mce1 *operon mutant has been shown to have an overabundance of free mycolic acids in the outer lipid layer (S. Cantrell, personal communication), supporting the proposition that *mce1 *and related operons play a role in remodeling the cell envelope. The presence of *mce *operons in Gram-negative bacteria and *Actinomycetales *genera that possess a somewhat analogous outer lipid bilayer raises the possiblity that the *mce *operons are involved in maintenance of outer membrane integrity. However, their presence in other *Actinomycetales *with typical Gram-positive type cell envelopes appears to preclude this hypothesis. In addition, the absence of *mce *operons in *Corynebacterium *species indicates that their function is not essential for maintenance of an outer lipid bilayer.

Based on a stated similarity of the ATPase component to GluA of *Corynebacterium glutamicum*, Meidanis *et al*. [62] proposed that the *Xylella fastidiosa mce*-like operon (XF0421-19) encoded a glutamate importer. It was subsequently shown that a mutation within the homologous *N*. *meningitidis gltT *operon resulted in impaired glutamate-specific uptake at low sodium concentrations [63]. Glutamate is a prominent constituent of peptidoglycan; thus, disruption of its uptake in the proteobacterial *mce *operon mutants could perhaps account for the observed effect on cell envelope properties. Also relevant in this respect, is the conserved location of the peptidoglycan biosynthetic gene, *murA*, downstream of the Mce transporter genes in γ-*Proteobacteria*.

Homologs of the Mkl, Mce and DUF140 proteins have also been identified in plants [64]. The *Arabidopsis *homologs of DUF140 (TGD1, At1g19800) and Mce (TGD2, At3g20320) both localize to the inner plastid membrane, with the Mce domain located in the intra-membrane space. Lipid binding studies demonstrated that TGD1 specifically bound 1,2-diacyl-*sn*-glycerol 3-phosphate (phosphatidic acid). TGD1 and TGD2 mutants exhibited identical phenotypes consistent with disruption of transport of ER-derived phosphatidic acid into chloroplasts, suggesting the TGD proteins form part of a lipid translocator [65-67].

Orthologous ABC transporters are expected to be functionally equivalent [13-15], thus the proposal of both phosphatidic acid and glutamate as possible substrates of the Mce transporters is puzzling. It is noteworthy that in sequence analyses, by us and others, the Mkl-like ATPases are not closely related to GluA [8]. If the bacterial Mce homologs have phospholipid binding function, equivalent to TGD1, this might enable interaction with host cell membranes and explain the invasive phenotype associated with the *mce *loci. It is generally accepted that host-derived lipids are the primary source of carbon utilized by *M. tuberculosis in vivo *[68]; however no mechanism of lipid import has been identified. Thus it is enticing to hypothesize that the Mce transporters might perform this role. Inclusion of the fatty-acyl CoA synthetase, *fadD5*, in the *mce1 *operon and repression of the operon by a FadR-like regulator, lends some support to this conjecture [45].

The canonical eight-gene *mce *operon has undergone extensive proliferation and deletion events within certain *Actinomycetales *lineages, most notably in *Mycobacterium *and *Nocardia *species. The simplest explanation for the presence of multiple *mce *operons is that it facilitates elevated expression. However, evidence from transcriptional analyses of *M. tuberculosis *suggest that, at least in this organism, the operons are not co-regulated [69-72]; in addition, three of the four operons are associated with transcriptional regulators [45,73]. In competitive mouse infections, Sassetti and Rubin [6] found that an *mce1 *mutant exhibited a growth defect during the first 1–2 weeks of infection, whilst an *mce4 *mutant showed attenuation 3–4 weeks after inoculation. These observations support the proposition that the operons function at different stages of infection. Differential expression of the individual Mce transporters may reflect optimization for substrate uptake under differing conditions, such as in the low sodium intracellular environment; alternatively, they might have varying substrate specificities.

The number of *mce *operons in individual species appears to reflect the variety of environmental niches inhabited. Thus, the fast-growing, typically soil-dwelling, *Mycobacterium *species possess the greatest number, with polycyclic aromatic hydrocarbon-degrading species, isolated from bioremediation sites, containing the most [74]. In contrast, the host-specialized, slow-growing pathogenic species possess fewer operons, and the obligate intracellular pathogen, *M. leprae*, encodes a single complete *mce *operon. A high degree of sequence similarity indicates that the *mce1 *operon duplicated to create *mce2 *relatively recently. In *M. tuberculosis *complex strains, *mce *frameshift mutations are found conspicuously in these two operons: of the five described in this paper, four are in *mce2 *and the fifth is in *mce1*. This pattern may reflect the functional divergence of the *mce1 *and *mce2 *operons.

With the exception of mycolic acids, the distribution of morphological and chemotaxonomic traits within the *Actinomycetales *is polyphyletic [75]. Given the incongruent taxonomic distribution of the *mce *operons and their proposed role in integrity of the cell envelope, it is pertinent to note that presence of *mce *operons does not correlate with type of peptidoglycan, menaquinones, phospholipids or fatty acids in the cell envelope [75,76]. In addition, there is no correlation with oxygen requirement, habitat or pathogenicity.

## Conclusion

The available evidence suggests that the *mce *operons encode a novel subfamily of ABC transporter uptake systems comprised of DUF140 permease components, Mce-like substrate-binding proteins, and Mkl-type ATPase domains. Disruption of *mce *operons, in both *Actinomycetales *and Gram-negative bacteria, affects properties of the cell envelope and associated virulence phenotypes of pathogenic species. Empirical studies have implicated both glutamate and phosphatidic acid as substrates of *mce*-like transporters; thus, although the precise substrate specificity of the *M. tuberculosis *Mce transporters remains uncertain, we conclude that it is likely to be an organic acid precursor of cell envelope biogenesis.

## Methods

### Databases

Gene annotations and protein sequences were obtained from the publicly available databases: UniProt [77,78]; TIGR Comprehensive Microbial Resource (CMR) [79,80]; NCBI Microbial Genome Project [81]; Joint Genome Institute Microbial Genomics Database [82]; and TubercuList [44]. Sequences are referred to by the ordered locus name provided in these databases. Protein classification was informed by interrogation of conserved domain and motif databases: InterPro (IPR) [26,83], Pfam (PF) [27,31], TIGRFAM (TIGR) [28,79], and PROSITE (PS) [84,85]. The ABC transporter classification database, ABCISSE, was also consulted [29].

### BLAST analyses

Sequence similarity searches were performed by BLASTP against complete microbial genome sequences deposited in the TIGR-CMR and NCBI Microbial Genome Project databases [79,81,86]. To determine whether the EExDA motif identified in YrbE proteins was uniquely characteristic of the DUF140 family, we performed a BLASTP search of NCBI Microbial Genome Project with the *Actinomycetales *YrbE consensus motif (PLVTGLALAGAGGAAITADLGARRIREEIDALEVMGIDPISRLVVPR) using the default parameters, except with no filter and expect threshold of 100. To identify homologs of the *M. tuberculosis *Mas domain, each of the eight sequences was used in a PSI-BLAST query against the NCBI non-redundant database [87]. We used an inclusion threshold of P < 10^-5 ^and the scores were adjusted with composition-based statistics; these parameters resulted in convergence after 6–8 iterations.

### Multiple alignment and phylogenetic analyses

Phylogenetic analyses were conducted using the MEGA version 3.1 suite of programs [88]. Multiple alignments were constructed by CLUSTAL-W using the Gonnet weight matrix and default gap penalties [89]. Unrooted trees were computed by the neighbor-joining method. The consensus tree, after 500 bootstrap replicates, was displayed graphically with Tree Explorer. In addition, CLUSTAL-W alignments were converted to PHYLIP format and trees computed by the maximum likelihood method implemented by PROML using default parameters [90]. In all cases this resulted in a tree with topology that was essentially the same as the neighbor-joining tree generated by MEGA. Percentage pairwise similarity scores were calculated by CLUSTAL-X [91].

### Identification of conserved motifs

The MEME server was used to discover highly conserved sequence motifs within groups of homologous proteins [92,93]. Motifs were displayed graphically using WebLogo [94,95].

### Secondary structure and topology prediction

Groups of aligned orthologs were submitted to JPred [96], a consensus secondary structure prediction server, that provides improved accuracy over single sequence prediction methods [97]. Comparison of predictions between orthologous clusters by visual inspection allowed estimation of the consensus structure for a homologous family. Coiled-coils were predicted using the Lupas COILS algorithm through the JPred server [98].

Protein sequences were analyzed by SignalP and TatP to identify Sec- and Tat-dependent signal sequences [38,39,99]. The reliability of prediction of transmembrane helices and topology of proteins increases when different methods are combined [100]. Hence, we submitted sequences to TMHMM [101,102], HMMTOP [103,104] and TopPred [105,106], and determined the consensus prediction by manual comparison.

## Authors' contributions

NC conceived, designed and performed the study. LWR helped to interpret the data. NC drafted the manuscript; both authors read and approved the final manuscript.

## Figures and Tables

**Table 1:** Distribution of Mce and YrbE proteins within the Order *Actinomycetales*^a^

**Suborder**	**Family**	**Species**	**Mce**^**b**^	**DUF140**^**c**^	**Source**
*Actinomycinaeae*	*Actinomycetaceae*	*Actinomyces naeslundii *MG1	0	0	UniProt
*Corynebacterineae*	*Corynebacteriaceae*	*Corynebacterium diphtheriae *NCTC 13129	0	0	UniProt
		*Corynebacterium efficiens *YS-314	0	0	UniProt
		*Corynebacterium glutamicum *ATCC 13032	0	0	UniProt
		*Corynebacterium jeikeium *K411	0	0	UniProt
	*Mycobacteriaceae*	*Mycobacterium leprae *TN	6	2	UniProt
		*Mycobacterium bovis *AF2122/97	18	7	UniProt
		*Mycobacterium tuberculosis *CDC1551	24	7	TIGR
		*Mycobacterium tuberculosis *H37Rv	24	8	TIGR
		*Mycobacterium paratuberculosis *K-10	48	14	UniProt
		*Mycobacterium smegmatis *MC2 155	34	11	TIGR
		*Mycobacterium *sp. MCS	38	11	JGI
		*Mycobacterium *sp. KMS	38	12	JGI
		*Mycobacterium *sp. JLS	50	16	JGI
		*Mycobacterium flavescens *PYR-GCK	48	13	UniProt
		*Mycobacterium vanbaalenii *PYR-1	66	24	UniProt
	*Nocardiaceae*	*Nocardia farcinica *IFM 10152	36	12	UniProt
*Frankineae*	*Acidothermaceae*	*Acidothermus cellulolyticus *11B	0	0	UniProt
	*Frankiaceae*	*Frankia *sp. CcI3	0	0	UniProt
		*Frankia *sp. EAN1pec	0	0	UniProt
	*Kineosporiaceae*	*Kineococcus radiotolerans *SRS30216	0	0	UniProt
*Micrococcineae*	*Brevibacteriaceae*	*Brevibacterium linens *BL2	0	0	JGI
	*Cellulomonadaceae*	*Tropheryma whipplei *str. Twist	0	0	UniProt
		*Tropheryma whipplei *TW08/27	0	0	UniProt
	*Intrasporangiaceae*	*Janibacter *sp. HTCC2649	6	2	NCBI
	*Microbacteriaceae*	*Leifsonia xyli *subsp.*xyli *str. CTCB07	0	0	UniProt
	*Micrococcaceae*	*Arthrobacter aurescens *TC1	0	0	UniProt
		*Arthrobacter *sp. FB24	0	0	UniProt
*Propionibacterineae*	*Nocardioidaceae*	*Nocardioides *sp. JS614	12	3	UniProt
	*Propionibacteriaceae*	*Propionibacterium acnes *KPA171202	0	0	UniProt
*Pseudonocardineae*	*Pseudonocardiaceae*	*Amycolatopsis mediterranei *^d^	6	2	Pfam
*Streptomycineae*	*Streptomycetaceae*	*Streptomyces avermitilis *MA-4680	6	2	UniProt
		*Streptomyces coelicolor *A3(2)	6	2	UniProt
*Streptosporangineae*	*Nocardiopsaceae*	*Thermobifida fusca *YX	0	0	UniProt

^a ^Taxonomy from Bergey's Manual of Systematic Bacteriology [107]

^b ^Number of proteins classified as PF02470

^c ^Number of proteins classified as PF02405

^d ^Incomplete genome, EMBL Accession AF040570

**Table 2:** *Actinomycetales mce*-linked ATPases and mycobacterial orthologs

**Organism**	**ATPase**
*Amycolatopsis mediterranei*	TrEMBL: Q7BUF5
*Janibacter *sp. HTCC2649	JNB_08429
*Nocardia farcinica*	nfa51100
*Nocardioides *sp. JS614	NocaDRAFT_4321
*Streptomyces avermitilis*	SAV5902
*Streptomyces coelicolor*	SCO2422
*Mycobacterium bovis*	Mb0674
*Mycobacterium flavescens*	MflvDRAFT_3283
*Mycobacterium leprae*	ML1892
*Mycobacterium paratuberculosis*	MAP4129
*Mycobacterium smegmatis*	MSMEG1359
*Mycobacterium *sp. JLS	MjlsDRAFT_1757
*Mycobacterium *sp. KMS	MkmsDRAFT_1059
*Mycobacterium *sp. MCS	MmcsDRAFT_0968
*Mycobacterium tuberculosis *CDC1551	MT0684
*Mycobacterium tuberculosis *H37Rv	Rv0655
*Mycobacterium vanbaalenii*	MvanDRAFT_5200

**Table 3:** Classification of *Actinomycetales yrbE *and *mce *genes ^a^

**Prefix**^**b**^	***yrbE1A***	***yrbE1B***	***mce1A***	***mce1B***	***mce1C***	***mce1D***	***mce1E***	***mce1F***
**Rv**	0167	0168	0169	0170	0171	0172	0173	0174
**MT**	0176	0177r^c^	0178	0179	0180	0181	0182	0183
**Mb**	0173	0174	0175	0176	0177	0178	0179	0180
**ML**	2587	2588	2589	2590	2591	2592	2593	2594
**MAP**	3602	3603	3604	3605	3606	3607	3608	3609
**MSMEG**	0126	0127	0128	0129	0130	0131	0132	0133

	***yrbE2A***	***yrbE2B***	***mce2A***	***mce2B***	***mce2C***	***mce2D***	***mce2E***	***mce2F***
**Rv**	0587	0588	0589	0590	0591	0592	0593	0594
**MT**	0616	0617	0618	0619	0621	0622	0623	0624
**Mb**	0602	0603	0604	0605	0606	0607	0609	0610
**MAP**	4082	4083	4084	4085	4086	4087	4088	4089

	***yrbE3A***	***yrbE3B***	***mce3A***	***mce3B***	***mce3C***	***mce3D***	***mce3E***	***mce3F***
**Rv**	1964	1965	1966	1967	1968	1969	1970	1971
**MT**	2016	2017	2018	2019	2020	2021	2022	2023
**Mb**	1999							
**MAP**	2117c	2117c.1^d^	2116c	2115c	2114c	2113c	2112c	2111c
**MSMEG**	0335	0336^e^	0337	0338	0339	0340	0341	0342

	***yrbE4A***	***yrbE4B***	***mce4A***	***mce4B***	***mce4C***	***mce4D***	***mce4E***	***mce4F***
**Rv**	3451c	3450c	3499c	3498c	3497c	3496c	3495c	3494c
**MT**	3605	3604	3603	3602	3601	3600	3599	3598
**Mb**	3531c	3530c	3529c	3528c	3527c	3526c	3525c	3524c
**MAP**	0562	0563	0564	0565	0566	0567	0568	0569
**MSMEG**	5861	5860	5859.3^e^	5859.2^e^	5859.1^e^	5859	5858	5857.1^e^
**nfa**	5350	5360	5370	5380	5390	5400	5410	5420

	***yrbE5A***	***yrbE5B***	***mce5A***	***mce5B***	***mce5C***	***mce5D***	***mce5E***	***mce5F***
**MAP**^**f**^	0757	0758	0759	0760	0761	0762/3^g^	0764	0765
**MAP**			2189	2190	2191	2192	2193^h^	2194
**MSMEG**	2855	2856	2857	2858	2859	2860	2861	2862
**MSMEG**^**f**^	4785	4784	4783	4782	me^i^	4777	4776	4775

	***yrbE6A***	***yrbE6B***	***mce6A***	***mce6B***	***mce6C***	***mce6D***	***mce6E***	***mce6F***
**nfa**	51090	51080	51070	51060	51050	51040	51030	51020
**SCO**	5901	5900	5899	5898	5897	5896	5895	5894
**SAV**	2421	2420	2419	2418	2417	2416	2415	2514

	***yrbE7A***	***yrbE7B***	***mce7A***	***mce7B***	***mce7C***	***mce7D***	***mce7E***	***mce7F***
**MAP**^**j**^	me^i^	0107	0108	0109	0110	0111	0112	0113
**MAP**	1849	1850	1851	1852	1853	1854	1855	1856
**MSMEG**^**j**^	1131	1132	1133	1134	1135	1136	1137	1138
**nfa**	50540	50530	50520	50510	50500	50490	50480	50470
**nfa**	56330	56320	56310	56300	56290	56280	56270	56260

	***yrbE8A***	***yrbE8B***	***mce8A***	***mce8B***	***mce8C***	***mce8D***	***mce8E***	***mce8F***
**nfa**	11130	11140	11150	11160	11170	11180	11190	11200
**nfa**	29780	29770	29760^h^	29750	29740	29730	29720	29710

^a ^Operons *mce1-4 *designated as in TubercuList; *mce5*-*8 *designated herein. Gene names in organisms other than *M. tuberculosis *do not correspond to those given in genome annotation.

^b ^Organism specific gene number prefix: Rv, *M. tuberculosis *H37Rv; MT, *M. tuberculosis *CDC1551; Mb, *M. bovis*; ML, *M. leprae*; MAP, *M. paratuberculosis*; MSMEG, *M. smegmatis*; nfa, *N. farcinica*; SCO, *S. coelicolor*; SAV, *S. avermitilis*.

^c ^Orthologous sequence present, but ORF annotated in reverse direction.

^d ^Orthologous sequence present, but not annotated. ORF extends ~400 bp at 5'end.

^e ^Orthologous sequence present, but not annotated.

^f ^Orthology inferred from synteny.

^g ^Contains frameshift mutation, resulting in two ORFs.

^h ^Not a member of IPR003399 or IPR005693.

^i ^Insertion of mobile element.

^j ^Orthology inferred from synteny.

**Table 4:** Mas Homologs in Selected *Actinomycetales *Genomes^ab^

	**Rv**	**ML**	**MAP**	**MSMEG**	**nfa**	**SAV**	**SCO**
**Mas1 A**	0175 (213)	2595 (182)	3610 (213)	0134 (202)			
**B**	0176 (322)	2596 (325)	3611 (323)	0135 (288)			
**C**	0177 (184)	2597 (184)	3612 (184)	0136 (182)			
**D**	0178 (244)	2598 (184)	3613 (252)	0137 (296)			

**Mas3 A**	1972 (191)		2110c (203)	0343 (200)			
**B**	1973 (160)		2109/7c^c^	0344 (202)			

**Mas4 A**	3493c (242)		0570 (243)	5857 (233)	5430 (315)		
**B**	3492c (160)		0571 (164)	5856 (161)	5440 (162)		

**Mas6 A**					51010 (248)	5893 (177)	2413 (170)
**B**					51000 (274)	5892 (272)	2412 (219)
**C**							2411 (184)
**D**							2410 (253)

**Mas7-1 A**			0114 (198)	1139 (230)	50460 (246)		
**Mas7-2 A**			1857 (227)		56250 (321)		

**ClusterI A**	1363c (261)		0751c (295)	4759.2 (303)			
**B**	1362c (220)		0750c (187)	4759 (200)			
**A**			0768c (298)	2867 (190)			
**B**			0767c (224)	2868 (218)			

**ClusterII A**	0199 (219)	2614 (229)		0225 (206)	6070 (197)		
**B**	0200 (229)	2615 (224)		0226 (229)			

**A**	2390c (185)		0090c (212)				

**A**				0878 (167)			
**B**				0879 (496)			

**A**				5189 (231)			
**B**				5190 (192)			

^a ^Organism specific gene number prefix: Rv, *M. tuberculosis *H37Rv; ML, *M. leprae*; MAP, *M. paratuberculosis*; MSMEG, *M. smegmatis*; nfa, *N. farcinica*; SCO, *S. coelicolor*; SAV, *S. avermitilis*.

^b ^Each row contains putative orthologs. Length of protein in amino acids shown in parentheses.

^c ^ORF is interrupted by a transposase, MAP2108.
